# Supplementary material for: Resolving the full spectrum of human genome variation using Linked-Reads
Source: Genome Res. 2019 Apr;29(4):635–45. doi: 10.1101/gr.234443.118 (PMC6442396; doi:10.1101/gr.234443.118)
Supplement: Supplemental Material [file supp_gr.234443.118_Supplemental_longranger-2.2.zip › longranger-2.2.tar.bz2!/lr2.2-public/lib/stan/cmdstan/stan/doc/api/html/agrad_2fwd_2functions_2binary__log__loss_8hpp_source.html]

Stan: src/stan/agrad/fwd/functions/binary\_log\_loss.hpp Source File


|  |  |
| --- | --- |
|  | Stan  2.5.0  probability, sampling & optimization |


- Main Page
- Related Pages
- Namespaces
- Classes
- Files

- File List
- File Members

All Classes Namespaces Files Functions Variables Typedefs Enumerations Enumerator Friends Macros Pages

- src
- stan
- agrad
- fwd
- functions

binary\_log\_loss.hpp

Go to the documentation of this file.

1 #ifndef STAN\_\_AGRAD\_\_FWD\_\_FUNCTIONS\_\_BINARY\_LOG\_LOSS\_HPP

2 #define STAN\_\_AGRAD\_\_FWD\_\_FUNCTIONS\_\_BINARY\_LOG\_LOSS\_HPP

3

4 #include <stan/agrad/fwd/fvar.hpp>

5 #include <stan/meta/traits.hpp>

6 #include <stan/math/functions/binary\_log\_loss.hpp>

7

8 namespace stan {

9

10  namespace agrad {

11

12  template <typename T>

13  inline

14  fvar<T>

15  binary\_log\_loss(const int y, const fvar<T>& y\_hat) {

16  using stan::math::binary\_log\_loss;

17

18  if (y)

19  return fvar<T>(binary\_log\_loss(y,y\_hat.val\_), -y\_hat.d\_ / y\_hat.val\_);

20  else

21  return fvar<T>(binary\_log\_loss(y,y\_hat.val\_), y\_hat.d\_ / (1.0 - y\_hat.val\_));

22  }

23  }

24 }

25 #endif

stan::math::binary\_log\_loss

boost::math::tools::promote\_args< T >::type binary\_log\_loss(const int y, const T y\_hat)

Returns the log loss function for binary classification with specified reference and response values...

**Definition:** binary\_log\_loss.hpp:26

stan::agrad::fvar

**Definition:** fvar.hpp:13

traits.hpp

fvar.hpp

binary\_log\_loss.hpp

stan::agrad::fvar::d\_

T d\_

**Definition:** fvar.hpp:16

stan::agrad::binary\_log\_loss

fvar< T > binary\_log\_loss(const int y, const fvar< T > &y\_hat)

**Definition:** binary\_log\_loss.hpp:15

stan::agrad::fvar::val\_

T val\_

**Definition:** fvar.hpp:15


---

[ Stan Home Page ]

*© 2011–2014,
Stan Development Team.*
